# Supplementary material for: Multi-task adaptive deep sparse canonical correlation analysis for multi-omics cancer survival prediction
Source: PLoS One. 2026 Apr 13;21(4):e0346274. doi: 10.1371/journal.pone.0346274 (PMC13075707; doi:10.1371/journal.pone.0346274)
Supplement: S1 Table — Metric: concordance index (C-index). Outer CV = 10 folds; inner CV = 10 folds for tuning. Values are mean ± SD across outer folds. (DOCX) [file pone.0346274.s001.docx]

**Table S1. Nested cross-validation performance (outer test folds).**

*Metric: concordance index (C-index). Outer CV = 10 folds; inner CV = 10 folds for tuning. Values are mean ± SD across outer folds.*

| **Cohort** | **Method** | **Outer C-index (mean ± SD)** | **Median** | **IQR** |
| --- | --- | --- | --- | --- |
| BRCA | MT-ADSCCA | 0.7391 ± 0.018 | 0.7391 | 0.720–0.742 |
| BRCA | DeepSurv | 0.7087 ± 0.020 | 0.7087 | 0.691–0.716 |
| BRCA | MTLSA | 0.6894 ± 0.022 | 0.6894 | 0.672–0.701 |
| BRCA | LASSO-Cox | 0.6807 ± 0.013 | 0.6807 | 0.670–0.688 |
| BRCA | RSF | 0.6523 ± 0.024 | 0.6523 | 0.635–0.668 |
| GBMLGG | MT-ADSCCA | 0.8449 ± 0.012 | 0.8449 | 0.834–0.850 |
| GBMLGG | DeepSurv | 0.8056 ± 0.014 | 0.8056 | 0.797–0.816 |
| GBMLGG | MTLSA | 0.7817 ± 0.015 | 0.7817 | 0.771–0.790 |
| GBMLGG | LASSO-Cox | 0.7548 ± 0.016 | 0.7548 | 0.742–0.764 |
| GBMLGG | RSF | 0.7426 ± 0.018 | 0.7426 | 0.729–0.754 |
| KIPAN | MT-ADSCCA | 0.7812 ± 0.014 | 0.7812 | 0.767–0.786 |
| KIPAN | DeepSurv | 0.7578 ± 0.016 | 0.7578 | 0.743–0.766 |
| KIPAN | MTLSA | 0.7413 ± 0.014 | 0.7413 | 0.729–0.748 |
| KIPAN | LASSO-Cox | 0.7342 ± 0.021 | 0.7342 | 0.719–0.748 |
| KIPAN | RSF | 0.7221 ± 0.017 | 0.7221 | 0.709–0.733 |
