# Supplementary material for: Multi-task adaptive deep sparse canonical correlation analysis for multi-omics cancer survival prediction
Source: PLoS One. 2026 Apr 13;21(4):e0346274. doi: 10.1371/journal.pone.0346274 (PMC13075707; doi:10.1371/journal.pone.0346274)
Supplement: S2 Table — Genes were ordered by chromosome and genomic coordinate in the original model. In the shuffled setting, gene order was randomly permuted 20 times per fold; the table reports mean ± SD across all permutations and folds. (DOCX) [file pone.0346274.s002.docx]

**Table S2. Shuffle test for the genomically ordered BiLSTM–Cox survival head.**

*Genes were ordered by chromosome and genomic coordinate in the original model. In the shuffled setting, gene order was randomly permuted 20 times per fold; the table reports mean ± SD across all permutations and folds.*

| **Cohort** | **Setting** | **C-index (mean ± SD)** | **ΔC-index** | **Median log-rank p** |
| --- | --- | --- | --- | --- |
| BRCA | Original order | 0.739 ± 0.017 | — | 2.37e−05 |
| BRCA | Shuffled (20×) | 0.719 ± 0.018 | -2.8% | 0.031 |
| GBMLGG | Original order | 0.845 ± 0.013 | — | 6.40e−07 |
| GBMLGG | Shuffled (20×) | 0.800 ± 0.016 | -5.4% | 0.008 |
| KIPAN | Original order | 0.781 ± 0.013 | — | 4.95e−04 |
| KIPAN | Shuffled (20×) | 0.752 ± 0.015 | -3.9% | 0.017 |
