# Supplementary material for: Multi-task adaptive deep sparse canonical correlation analysis for multi-omics cancer survival prediction
Source: PLoS One. 2026 Apr 13;21(4):e0346274. doi: 10.1371/journal.pone.0346274 (PMC13075707; doi:10.1371/journal.pone.0346274)
Supplement: S3 Table — This configuration was fixed across cohorts and tuned within the inner cross-validation loop where applicable. (DOCX) [file pone.0346274.s003.docx]

**Table S3. DeepSurv baseline configuration used in this study.**

*This configuration was fixed across cohorts and tuned within the inner cross-validation loop where applicable.*

| **Item** | **Setting** |
| --- | --- |
| Input features | Concatenated multi-omics features (same feature set across baselines within each comparison) |
| Network | MLP with 2 hidden layers: (128, 64) |
| Activation | ReLU |
| Dropout | 0.20 |
| L2 weight decay | 1e−4 |
| Optimizer | Adam |
| Learning rate | 1e−3 |
| Batch size | 64 |
| Epochs | Up to 300 (early stopping enabled) |
| Early stopping | Patience = 20 epochs based on validation Cox loss |
| Random seeds | Fixed per fold for reproducibility |
