# Supplementary material for: Multi-task adaptive deep sparse canonical correlation analysis for multi-omics cancer survival prediction
Source: PLoS One. 2026 Apr 13;21(4):e0346274. doi: 10.1371/journal.pone.0346274 (PMC13075707; doi:10.1371/journal.pone.0346274)
Supplement: S4 Table — Latent representations from each method were evaluated using the same survival head for fairness. Statistical tests were performed on per-fold C-index values (paired Wilcoxon). Bonferroni correction was applied within each cohort. (DOCX) [file pone.0346274.s004.docx]

**Table S4. Additional multi-omics integration baselines under identical folds.**

*Latent representations from each method were evaluated using the same survival head for fairness. Statistical tests were performed on per-fold C-index values (paired Wilcoxon). Bonferroni correction was applied within each cohort.*

| **Cohort** | **Method** | **C-index (mean ± SD)** | **Δ vs MT-ADSCCA** | **Wilcoxon p** | **Bonferroni p** | **Cohen’s d** |
| --- | --- | --- | --- | --- | --- | --- |
| BRCA | MT-ADSCCA | 0.739 ± 0.017 | — | — | — | — |
| BRCA | DeepCCA + Cox | 0.712 ± 0.019 | −0.027 | 0.004 | 0.012 | 0.98 |
| BRCA | MOFA+ + Cox | 0.705 ± 0.020 | −0.034 | 0.002 | 0.006 | 1.15 |
| BRCA | RGCCA + Cox | 0.708 ± 0.018 | −0.031 | 0.003 | 0.009 | 1.05 |
