# Supplementary material for: Multi-task adaptive deep sparse canonical correlation analysis for multi-omics cancer survival prediction
Source: PLoS One. 2026 Apr 13;21(4):e0346274. doi: 10.1371/journal.pone.0346274 (PMC13075707; doi:10.1371/journal.pone.0346274)
Supplement: S5 Table — Values are mean ± SD across outer folds. Corr (zx1, zy1) denotes the canonical correlation between the first latent components learned from expression and methylation encoders. (DOCX) [file pone.0346274.s005.docx]

**Table S5. Canonical correlation of the first canonical pair (train vs validation).**

*Values are mean ± SD across outer folds. Corr(zx1, zy1) denotes the canonical correlation between the first latent components learned from expression and methylation encoders.*

| **Cohort** | **Train Corr (mean ± SD)** | **Validation Corr (mean ± SD)** | **Generalization gap** |
| --- | --- | --- | --- |
| BRCA | 0.618 ± 0.026 | 0.579 ± 0.028 | 0.039 |
| GBMLGG | 0.666 ± 0.021 | 0.637 ± 0.023 | 0.029 |
| KIPAN | 0.603 ± 0.025 | 0.563 ± 0.027 | 0.040 |
