# Supplementary material for: Multi-task adaptive deep sparse canonical correlation analysis for multi-omics cancer survival prediction
Source: PLoS One. 2026 Apr 13;21(4):e0346274. doi: 10.1371/journal.pone.0346274 (PMC13075707; doi:10.1371/journal.pone.0346274)
Supplement: Attachment — Submitted filename: Response_to_Reviewers.docx [file pone.0346274.s007.docx]

Title: Response to Reviewer Comments for “Multi-task Adaptive Deep Sparse Canonical Correlation Analysis for Multi-omics Cancer Survival Prediction”

Manuscript ID: PONE-D-25-29777

Authors: Yan Wang, Yuanyuan Wu, Jing Chen, Zimo Zou

Dear Editor and Reviewers,

We sincerely thank the reviewers for their careful reading of our manuscript and for providing constructive comments. We have revised the manuscript substantially to improve clarity, methodological rigor, and biological interpretability. Below we provide a point-by-point response. Reviewer comments are reproduced in bold, followed by our responses. Where appropriate, we indicate the location of changes in the revised manuscript.

# Response to Reviewer #1

**1. The manuscript requires substantial revisions before publication.**

Response: We substantially revised the manuscript, including a complete language edit, a reorganization of the Methods and Results, and a clearer presentation of the MT-ADSCCA framework. We also corrected and rebuilt Table 1, improved figure captions, and ensured consistent terminology throughout the manuscript.

**2. Comprehensive language editing is needed.**

Response: We performed a full language revision across the manuscript, correcting fragmented sentences, tense drift, and missing punctuation (including punctuation following displayed equations).

**3. The Introduction does not clearly state the research gap or contributions.**

Response: We rewrote the Introduction to explicitly describe limitations of classical sparse CCA and existing deep multi-view approaches in the context of cancer multi-omics survival modeling. We added a concise contributions paragraph enumerating the specific methodological innovations of MT-ADSCCA.

**4. Provide a rationale for restricting the analysis to two omics layers or demonstrate scalability.**

Response: We added an explicit rationale explaining that mRNA expression and DNA methylation provide the highest overlap of matched samples across TCGA cohorts, while adding additional modalities substantially reduces cohort size due to missingness. We also added a short paragraph describing how the formulation generalizes to more than two omics layers (multi-view extension).

**5. Clarify the connection between classical SCCA and the proposed multi-task architecture.**

Response: We revised the Methods to provide a clearer mapping between the classical CCA/SCCA objective and the MT-ADSCCA loss design. Each loss component is now described with its role (correlation maximization, sparsity, reconstruction consistency, and normalization) and how adaptive uncertainty weighting balances their gradients during training.

**6. Cohort selection criteria are insufficiently described.**

Response: We added a dedicated cohort inclusion and filtering description in Methods, specifying survival-time filtering, matched-omics requirements, and final sample sizes per cohort. Event and censoring counts are now reported in Table 1.

**7. Table 1 contains empty rows; mark missing values and improve caption.**

Response: We rebuilt Table 1 following the reviewer’s guidance, removed empty rows, marked unavailable variables as N/A, and expanded the caption to clarify variable availability.

**8. Figures are low-resolution; provide vector formats.**

Response: We regenerated figures for submission in high-quality vector formats (EPS) with consistent fonts and color-blind–safe palettes. The manuscript now references these updated figures.

**9. Results lack biological interpretation; identify key genes and pathways.**

Response: We added a new Results subsection, “Biological Interpretation of Canonical Features,” which interprets the first canonical variate using the actual selected genes and reports functional-category enrichment. We intentionally emphasize functional categories rather than forcing KEGG pathway labels, because the learned signatures are not dominated by classical cell-cycle/DNA-repair markers in our cohorts.

**10. Benchmarking is limited; include additional baselines and statistical significance.**

Response: We strengthened benchmarking by adding paired statistical testing (Wilcoxon signed-rank) across folds, multiple-comparison correction, and effect sizes. We expanded benchmarking by adding three additional multi-omics integration baselines (DeepCCA, MOFA+, and RGCCA) and provided fair, reproducible implementations under identical nested cross-validation folds.

**11. Provide robustness evidence (nested CV or external validation).**

Response: We strengthened robustness evidence by adopting leakage-free nested 10-fold cross-validation and reporting fold-wise performance variability (Supplementary Table S1). We also added a shuffle test to validate the genomic-ordering assumption of the BiLSTM survival head (Supplementary Table S2), and included full baseline hyperparameter settings for reproducibility (Supplementary Table S3).

**12. State whether code and scripts will be released.**

Response: We added a reproducibility statement indicating that code, preprocessing scripts, and hyperparameter settings will be released upon acceptance (or made available to reviewers upon request).

**13. Minor issues (typos, undefined abbreviations, equation punctuation).**

Response: We systematically corrected typographical errors, defined abbreviations at first use (including in figure captions), standardized equation punctuation, and performed a final consistency pass across terminology and cross-references.

# Response to Reviewer #2

**1. Please provide one sentence with the main aim, data, main methodological contribution, comparisons, and primary outcome.**

Response: We added a one-sentence statement in the Abstract and Introduction specifying: (i) TCGA cohorts and the two omics layers (mRNA and DNA methylation), (ii) the main methodological contribution (adaptive multi-task sparse CCA for feature selection coupled with a BiLSTM–Cox survival head), (iii) the primary outcome (C-index), and (iv) the primary goal (improved survival prediction) with interpretable biomarker discovery as a secondary objective.

**2. Please add a Discussion section that interprets results and notes limitations and next steps.**

Response: We rewrote the Discussion to include biological interpretation, clinical relevance, limitations (two-modality design, lack of external validation, computational cost), and future directions (multi-omics extension, alternative architectures, and external-cohort validation).

**3. Concern about leakage: feature selection should be fit only on training folds.**

Response: We corrected the evaluation pipeline so that all preprocessing, feature selection (including MT-ADSCCA), and hyperparameter tuning are performed strictly within the training fold of the outer cross-validation loop, and then applied to the held-out test fold without refitting. This is now explicitly stated in Methods.

**4. BiLSTM ordering: state ordering and include a shuffle test and/or non-LSTM baseline.**

Response: We clarified that genes are ordered by chromosome and genomic coordinate. We added a shuffle test showing consistent degradation in performance when gene order is randomized (reported in Supplementary Table S2), supporting that the BiLSTM head leverages meaningful genomic neighborhood structure rather than arbitrary ordering.

**5. Clarify what is “deep” in MT-ADSCCA.**

Response: We clarified in Methods that the correlation-learning stage uses nonlinear encoders to learn representations for CCA-style alignment, while the survival stage uses a BiLSTM–Cox network. We also adjusted wording to avoid confusion between the feature-selection stage and the survival head.

**6. Add paired tests and multiple-comparison correction for comparisons.**

Response: We added paired Wilcoxon signed-rank tests on per-fold C-index values, multiple-comparison correction, and effect sizes. These results are summarized in Supplementary Table S4 and referenced in the Results.

**7. Report events vs censored per cohort and confirm event-stratified folds.**

Response: We rebuilt Table 1 to include event and censoring counts and follow-up summaries. We also clarified that outer CV splits are event-stratified to preserve censoring proportions across folds.

**8. DeepSurv baseline description is incorrect; provide architecture details.**

Response: We corrected the description of DeepSurv as a nonlinear MLP trained with Cox partial-likelihood loss, and we added baseline configuration details (layer widths, dropout, regularization, early stopping) in Supplementary Table S3 to ensure fair and reproducible comparison.

**Minor comments (terminology consistency, number-at-risk tables, Table 1 cleanup, ΔC-index reporting, supplementary hyperparameters and per-fold gene lists).**

Response: We standardized terminology throughout, rebuilt Table 1, added number-at-risk tables to Kaplan–Meier plots, and expanded supplementary materials to include hyperparameter settings and additional evaluation details. Where relevant, we report absolute C-index differences and variability across folds.

**Closing Statement**

We again thank the reviewers for their valuable feedback. We believe the revised manuscript is substantially improved in clarity, methodological rigor, biological interpretability, and reproducibility, and we hope it is now suitable for publication.
